# Competition Thesis in Radiography and Electro-Therapy

**Published:** 1921-07-23

**Authors:** 


### The Society of Radiographers.

### COMPETITION THESIS IN RADIOGRAPHY AND ELECTRO-THERAPY.

The first general meeting of the Society of Radiographers was held at University College, Gower Street, on Wednesday, June 29, the President, Sir Archibald Reid, in the chair. The Society was formed with the object of promoting the science and to regulate the practice of radiography and to consider and discuss all subjects affecting it; and, so far as possible, to make improvements in the law relating to radiography, and to interchange ideas and information on matters affecting it and allied subjects. Since its foundation nine months ago one examination has been held, and Diplomas have been granted. None of the members of the Society are "Associate" members, and it is the hope and desire of the Council that all membership shall be by examination only. It is hoped, by the courtesy of the Institution of Electrical Engineers, to hold meetings in the Lecture Room of the Institution on the second Tuesday in the months of October and December 1921, and February and April 1922. It is the intention of the Council to endeavour to make the Society the main body in this branch of scientific work, to which the hospitals, and possibly the Government, can turn for authoritative information on all matters affecting radiography; and it is hoped that an Information Bureau may be formed with a view to the general improvement in the conditions of workers. The question of the hours of duty of radiographic workers is a vital one, and the Protection Committee now sitting is suggesting to the employers of radiographers and their assistants that one month's leave of absence be granted annually to this class of employee, together with two afternoons a week off duty, and that female workers in the radiographic department shall not be called upon to do duty in the wards whilst carrying out this work. The following ladies and gentlemen have been awarded the Diploma of the Society: Messrs. A. L. Stent, A. J. Walton, William Watson, G. MacLardie, J. J. C. Knecht, E. V. Harris, H. E. Hardwick, G. W. Gibson, G. E. F. Fisher, H. F. Cheese, R. L. Read, T. Azeal, E. Varden, E. A. H. Barham, J. W. Bridge, S. J. Alavoine, E. E. M. Alexander, W. E. Curtis, C. R. S. Copeland, H. J. Suggars, C. T. Stockham, W. E. Smith, C. L. Winch, G. R. Ilsley, A. L. Townsend, H. F. Smith, H. Dixon, J. R. Levey, A. Buchner, G. G. Blake, E. T. Coppin, W. Deacon, G. Stewart, A. Haigh, G. W. Howard, W. G. Kirkham, H. P. March, W. Roberts, A. Jolly, J. Jones, G. L. Stiles, H. Shirley, W. Warrad, J. Williams, R. G. Blackall, F. E. Doran, A. V. Forder, H. Henry, H. Turner, G. F. Westlake; the Misses C. R. Lowe, R. V. H. Large, M. F. Smith, G. C. Shimell, G. K. Towgood, P. Kerry, A. M. Ashwin, B. Lowe, B. Matthews.

### Conditions for Theses.

It is proposed to invite theses in (*a*) radiography and (*b*) electro-therapy from present members of the Society, for which two prizes of ten guineas each (one from Sir Archibald Reid and the other from the Council of Radiographers) will be awarded to the writers of the two best theses. Each thesis is to be about 5,000 words, and must be sent in to the Council by December 31, 1921. The two classes are:

(1) *Radiographic Prize.*—The best thesis in one of the following subjects (illustrated by not less than six nor more than twelve photographs): The technique of examination of (1) urinary tract and gall-bladder; (2) spine and pelvis; (3) cranium.

(2) *Electro-therapy Prize.*—The best thesis in one of the following subjects—(1) The theory of ionisation, illustrated by experimental evidence; (2) the different methods of producing *x*-rays for therapy; (3) the method of diathermy and its application. (It is quite permissible for one candidate to try for both presentations.)

